# Impact of the N501Y substitution of SARS-CoV-2 Spike on neutralizing monoclonal antibodies targeting diverse epitopes

**DOI:** 10.1186/s12985-021-01554-8

**Published:** 2021-04-28

**Authors:** Lin Cheng, Shuo Song, Bing Zhou, Xiangyang Ge, Jiazhen Yu, Mingxia Zhang, Bin Ju, Zheng Zhang

**Affiliations:** 1 Institute for Hepatology, National Clinical Research Center for Infectious Disease, Shenzhen Third People’s Hospital; The Second Affiliated Hospital, School of Medicine, Southern University of Science and Technology, Shenzhen, 518112 Guangdong Province China; 2 Shenzhen Research Center for Communicable Disease Diagnosis and Treatment of Chinese Academy of Medical Science, Shenzhen, 518112 Guangdong Province China; 3 Guangdong Key Laboratory for Anti-Infection Drug Quality Evaluation, Shenzhen, 518112 Guangdong Province China; 4 Shenzhen Bay Laboratory, Shenzhen, 518055 Guangdong Province China

**Keywords:** SARS-CoV-2, N501Y variant, Neutralizing activity, Binding kinetics, Monoclonal neutralizing antibody

## Abstract

The emergence and rapid spread of the B.1.1.7 lineage (VOC-202012/01) SARS-CoV-2 variant has aroused global concern. The N501Y substitution is the only mutation in the interface between the RBD of B.1.1.7 and ACE2, raising concerns that its recognition by neutralizing antibodies may be affected. Here, we assessed the neutralizing activity and binding affinity of a panel of 12 monoclonal antibodies against the wild type and N501Y mutant SARS-CoV-2 pseudovirus and RBD protein, respectively. We found that the neutralization activity and binding affinity of most detected antibodies (10 out of 12) were unaffected, although the N501Y substitution decreased the neutralizing and binding activities of CB6 and increased that of BD-23. These findings could be of value in the development of therapeutic antibodies.

**Supplementary Information:**

The online version contains supplementary material available at 10.1186/s12985-021-01554-8.

## Introduction

The coronavirus disease 2019 (COVID-19) caused by severe acute respiratory syndrome coronavirus 2 (SARS-CoV-2) has emerged in late 2019 and lasted more than a year all over the world. Neutralizing antibodies (nAbs) could block the entry of SARS-CoV-2 into host cells by disturbing the interaction of viral spike with the cellular receptor, angiotensin-converting enzyme 2 (ACE2). So far, a large number of RBD-specific nAbs have been identified from convalescent individuals and immunized animals, some of which are promising candidates for treating and preventing COVID-19 and are undergoing clinical trials [1–5].

Given that SARS-CoV-2 is a single-stranded RNA virus, mutation could easily occur and accumulate in the process of the COVID-19 pandemic. Indeed, a novel viral variant recently emerged in England, named N501Y.V1 (also known as VOC-202012/01 or B.1.1.7 lineage), which is up to 70% more transmissible [6]. There are seven substitutions (N501Y, A570D, D614G, P681H, T716I, S982A, and D1118H) and three deletions (H69Del, V70Del, and Y144Del) in the spike of the N501Y.V1 variant comparing with the Wuhan-Hu-1 strain (wide type), with N501Y the only mutation in the ACE2 interface of the receptor binding domain (RBD). In addition, N501Y was also shared by another SARS-CoV-2 variant-N501Y.V2 reported from South Africa, also known as B.1.351 lineage containing three mutations (K417N, E484K, and N501Y) in the RBD [7].

Therefore, it is crucial to test and monitor the neutralizing sensibilities of emerging SARS-CoV-2 variants to the published nAbs especially which are undergoing clinical trials and good candidates for treating and preventing COVID-19. Currently, some researchers have focused on the analysis of viral variants escaping the neutralization of monoclonal nAbs isolated by themselves or published by others and polyclonal nAbs of sera samples from convalescent patients or vaccinated individuals [8–10]. The N501Y.V1 variant usually maintained or partially affected the neutralizing sensitivity to most of nAbs, but N501Y.V2 could fully escaped from the neutralization of certain types of nAbs, which become a serious challenge to the current antibody and vaccine candidates.

In this study, we further combined a RBD-specific monoclonal nAbs panel involving twelve published antibodies from different classes with diverse neutralizing epitopes, and measured their neutralizations and binding affinities against the wild type and N501Y mutant SARS-CoV-2, which will enrich the research in the field of viral escape and be crucial to the control of COVID-19.

## Materials and methods

### The expression and purification of monoclonal neutralizing antibodies

Gene sequences of published nAbs downloaded from the National Center of Biotechnology Information (NCBI) were synthesized and cloned into the human full-length IgG1 expression vectors (Sangon Biotech, Shanghai). Paired heavy and light chains were co-transfected into 293 F cells, and antibodies were purified from cell supernatants using protein A columns according to the manufacturer’s instructions (National Engineering Research Center for Biotechnology, Beijing) after five days. Purified nAbs were quantified using a NanoDrop spectrophotometer and stored at 4 °C.

### SARS-CoV-2 pseudovirus neutralization assay

SARS-CoV-2 pseudotyped-virus was generated by co-transfection of HEK-293T cells in T75 flask with 10 μg of SARS-CoV-2 spike-expressing plasmid and 20 μg of an env-deficient HIV-1 backbone vector (pNL4-3.Luc.R-E-). Two days post-transfection, pseudovirus-containing culture supernatant was harvested, clarified by centrifugation, filtered and then stored at −80 °C in 1.5-ml aliquots. To determine the neutralizing activity, serially diluted monoclonal antibodies were incubated with equal volume of diluted pseudovirus at 37 °C for 1 h. The antibody-virus mixtures were subsequently added into pre-seeded HEK-293T-hACE2 cells in duplicate. After a 48-h incubation, culture medium was removed and 100 μl of the Bright-Lite Luciferase reagent (Vazyme Biotech, Nanjing, China) was added to the cells. After a 2-min incubation at room temperature, 90 μl of cell lysate was transferred to 96-well white solid plates for measurements of luminescence using the Varioskan™ LUX multimode microplate reader (Thermo Fisher Scientific). The 50% inhibitory concentrations (IC_50_) was calculated using GraphPad Prism software by log (inhibitor) vs. normalized response—Variable slope (four parameters) model and fourfold of IC_50_ was set as the cutoff of significant change [11].

### Binding analysis by surface plasmon resonance (SPR)

The binding assays of monoclonal antibodies to the wild type and N501Y mutant SARS-CoV-2 RBDs were performed using the Biacore 8 K system (GE Healthcare). Specifically, one flow cell of the CM5 sensor chips were covalently coated with the wild type or N501Y mutant RBDs (Sino Biological, Beijing) in 10 mM sodium acetate buffer (pH 5.0) for a final RU (response units) around 250, whereas the other flow cell was left uncoated and blocked as a control. All the assays were run at a flow rate of 30 µl/min in HBS-EP buffer (10 mM HEPES pH 7.4, 150 mM NaCl, 3 mM EDTA and 0.05% Tween-20). Serially diluted antibodies were injected for 60 s respectively and the resulting data were fit in a 1:1 binding model with Biacore Evaluation software (GE Healthcare). Every measurement was performed three times and the individual values were used to produce the mean affinity constant and standard deviation.

### Main text

According to the competition with ACE2 and the accessibility of neutralizing epitopes on the RBD in ‘up’ or ‘down’ conformations, RBD-specific nAbs were classified into four classes[12]. As shown in Additional file 1: Fig. S1, nAbs of Class 1 and Class 2 bind to the receptor-binding motif (RBM) directly and near-RBM region on the RBD of spike, respectively, both of which could disturb the interaction between the RBD of virus and the cell receptor-ACE2. The nAbs of Class 3 and Class 4 do not compete with ACE2 directly, bind to the two sides of the base of RBD away from RBM, and usually cross-react with SARS-CoV. In this study, the twelve-nAb panel contains 3 nAbs in Class 1 (CB6, P2C-1F11, and REGN10933), 4 nAbs in Class 2 (BD-23, BD-368-2, P2B-2F6, and P2C-1A3), 2 nAbs in Class 3 (S309 and REGN10987), 1 nAb in Class 4 (EY6A), and 2 unclassified nAbs (COV2-2196 and CV07-287). To assess the effect of the N501Y substitution on the recognition with nAbs, we measured the neutralizations and binding affinities of these nAbs against the Wuhan-Hu-1 strain (WT) and N501Y mutant by pseudovirus neutralizing assay and surface plasmon resonance (SPR).

**Fig. 1:**
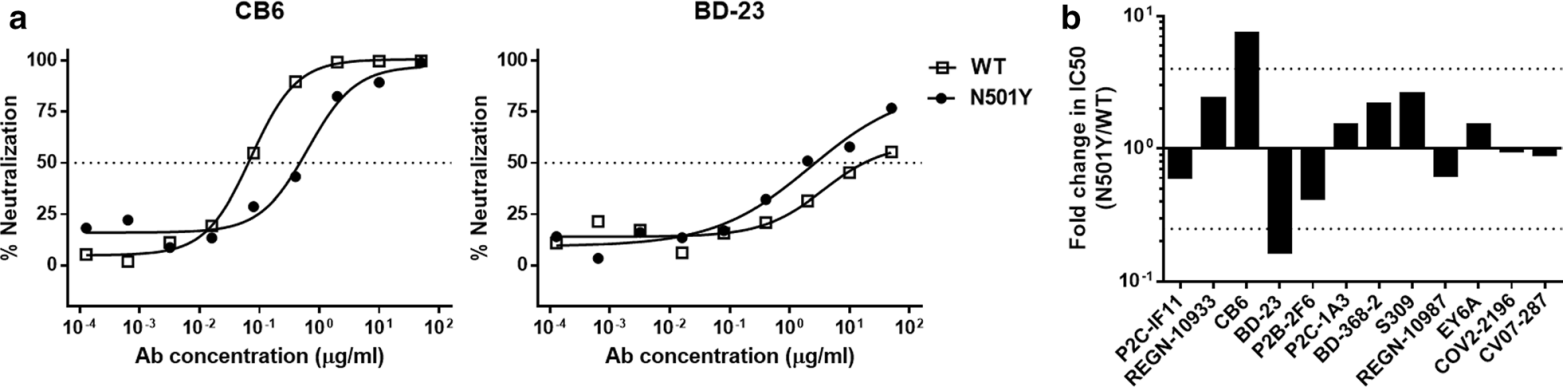
Neutralization of SARS-CoV-2 pseudovirus by the panel of monoclonal antibodies. (**a**) Examples of neutralization of indicated mAbs against pseudovirus bearing the spike of Wuhan strain (WT) or the N501Y mutant. (**b**) Summary of the data from the indicated panel of mAbs, with the values presented as mean of two independent experiments. The horizontal dashed lines indicate the threshold of fourfold difference

**Fig. 2:**
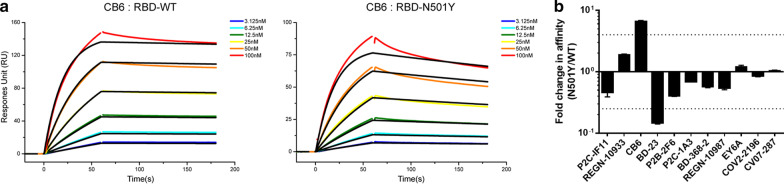
The binding affinities of the nAb panel to the RBD by SPR. (**a**) Examples of binding affinities of CB6 to spike RBD of Wuhan strain (WT) or the N501Y mutant. (**b**) Summary of the binding affinity data from the indicated mAbs, with the values presented as mean ± SD of three independent experiments. The horizontal dashed lines indicate the threshold of fourfold difference

Class 1 nAbs directly bound to the ACE2-binding site on the RBD, which shared the largest number of residues between their epitopes and ACE2-binding site. As shown in Table 1, the neutralizing activity and binding affinity of CB6 against N501Y were decreased distinctly compared to WT (7.38- and 6.67-fold, respectively), with another two Class 1 nAbs (P2C-1F11 and REGN10933) slightly affected (Figs. 1, 2, Table 1, Additional file 1: Fig. S2, Fig. S3). Reasonably, previous structural analysis of the CB6/RBD complex revealed that the epitope residue N501 contacted directly with CB6 but not with P2C-1F11 and REGN10933 [1, 3, 13]. Additionally, the free energy perturbation method also predicted that N501Y weaken the binding affinity of CB6 to RBD [14].

**Table 1:** Neutralizing activity and binding kinetics of nAbs against the wild type and N501Y mutant SARS-CoV-2

Antibody	Group^a^	Contact with N501	Structure	References	Neutralization (IC_50_, µg/ml, mean)^b^			Affinity (K_D_, nM, mean)^c^		
					WT	N501Y	FC^d^	WT	N501Y	FC^d^
P2C-1F11	Class 1	No	PDB: 7CDI	Ge et al. [13]	0.0300	0.0180	0.60	0.526	0.241	0.46
REGN10933	Class 1	No	PDB: 6XDG	Hansen, et al.[3]	0.0015	0.0035	2.36	0.0645	0.123	1.91
CB6	Class 1	Yes	PDB: 7C01	Shi et al. [1]	0.0456	0.3363	7.38	0.321	2.14	6.67
BD-23	Class 2	Yes	PDB: 7BYR	Cao et al. [21]	16.3052	2.4771	0.15	137	20.6	0.15
P2B-2F6	Class 2	No	PDB: 7BWJ	Ju et al. [2]	0.1644	0.0697	0.42	0.667	0.267	0.40
P2C-1A3	Class 2	No	PDB: 7CDJ	Ge et al. [13]	0.1862	0.2833	1.52	0.247	0.167	0.68
BD-368–2	Class 2	No	PDB: 7CHH	Du et al. [15]	0.0004	0.0008	2.11	0.0649	0.0368	0.57
S309	Class 3	No	PDB: 6WPS	Pinto et al. [17]	0.1641	0.4307	2.62	< 0.001	< 0.001	n.a.^e^
REGN10987	Class 3	No	PDB: 6XDG	Hansen, et al.[3]	0.0062	0.0039	0.63	0.108	0.0587	0.54
EY6A	Class 4	No	PDB: 6ZDH	Zhou et al. [16]	7.0680	10.7957	1.53	0.111	0.134	1.21
COV2-2196	n.a	n.a	n.a	Zost et al. [4]	0.0032	0.0029	0.93	0.0681	0.0581	0.85
CV07-287	n.a	n.a	n.a	Kreye et al. [5]	0.2930	0.2610	0.89	1.19	1.25	1.05

^a^Antibodies were classified into four groups based on their competitions with ACE2 and recognitions with ‘up’/‘down’ conformations of RBD [12]

^b^Neutralization was measured as IC_50_ in µg/ml of nAbs against SARS-CoV-2 pseudovirus

^c^Affinity was measured as K_D_ in nM of nAbs binding to SARS-CoV-2 RBD by SPR

^d^FC, Fold change. N501Y/WT

^e^n.a., not available

The Class 2 nAbs shared less or none overlapping residues with ACE2-binding site, but still showed strong competitions with ACE2. P2B-2F6 and P2C-1A3 both recognized an overlapped epitope on RBD yet with distinct angles of approach compared to P2C-1F11 (Class 1) [13]. BD-368-2 recognized RBD with a similar angle as P2B-2F6, and both inhibited the viral entry by a clash between the light chain and ACE2 [15]. These three nAbs of Class 2 did not contact with N501 epitope directly, so kept stable neutralizing and binding activities against N501Y mutant (Table 1, Additional file 1: Fig. S2, Fig. S3). Unexpectedly, the N501Y mutant virus was more sensitive (6.58-fold) to the neutralization of BD-23, another Class 2 antibody which also contact directly with N501 (Fig. 1, Table 1). Consistently, surface plasmon resonance analysis also revealed that the binding affinity of BD-23 to N501Y variant was increased 6.65-fold as compared with the wild type RBD (Fig. 2, Table 1, Additional file 1: Fig. S3).

Another two classes of nAbs recognized two completely distinct epitopes away from the ACE2-binding site. S309 (Class 3) bound both ‘up’ and ‘down’ states of the RBD, whereas EY6A (Class 4) only recognized the RBD epitope in up conformation [12]. Targeting the relatively conserved epitopes, S309 and EY6A both cross-neutralized SARS-CoV-2 and SARS-CoV. Seventeen out of 22 epitope residues recognized by S309 were conserved between SARS-CoV-2 and SARS-CoV, and 21 of 31 residues in the interaction between EY6A and RBD were conserved with a SARS-CoV specific nAb (CR3022) [16, 17]. Not surprisingly, the N501Y mutation did not affect the neutralizing and binding activities of S309 and EY6A (Figs. 1, 2, Table 1, Additional file 1: Fig. S2, Fig. S3).

The mutation N501Y in the circulating strain in England is not the first time identified from SARS-CoV-2 live virus. A previous study reported that N501Y existed in a mouse-adapted strain, named MASCp6, which was isolated by serial passaging of SARS-CoV-2 in the respiratory tract of mice and led to interstitial pneumonia in this mouse challenge model. Structural remodeling showed that the binding capacity of RBD of viral spike to mouse ACE2 protein was increased by the substitution of N501Y [18]. Another study developed a novel strain-MASCp36 by passaging of SARS-CoV-2 from MASCp6, which caused 100% fatality in aged mice. Sequence analysis revealed that another two amino acid substitutions (K417N and Q493H) appeared in RBD of SARS-CoV-2 spike besides N501Y. These mutations contributed the enhancement of binding affinity between RBD and mouse ACE2 which has been certified in vitro by surface plasmon resonance and structure analysis [19].

Indeed, it is reported that the neutralizing activities of four nAbs (lacking analysis of neutralizing epitope) against SARS-CoV-2_N501Y, and one nAb (03-1F9) showed a decrease of six-fold in the neutralization [20]. In addition, the K417N and E484K contribute more than a single N501Y substitution in the resistance of virus to the neutralization by nAbs in a study which measured the neutralizing potencies of eight human nAbs against two SARS-CoV-2 variants both carrying N501Y mutation (N501Y.V1 and N501Y.V2) [8]. Our results were similar with that of above two studies, which indicated that the N501Y mutation may partially affect the neutralizing potencies of some nAbs. Furthermore, a study analyzed the neutralization of SARS-CoV-2 variants by several classes of nAbs based on their different epitopes [10]. Consistently, we also revealed that current mutations on the RBD mainly influenced the neutralizations of nAbs targeting directly to the RBM and near-RBM region. Like S309 and EY6A, some nAbs bind the base of RBD and recognize the epitopes away from the RBM, whose neutralizations are barely affected by the current mutations of SARS-CoV-2. These results also explained the phenomenon that though the neutralization geometric mean titers of sera samples whether from convalescent patients or vaccinated individuals indeed reduced in different levels, a part of sera samples as a mixture of polyclonal nAbs with diverse epitopes still maintained the neutralizing activities against SARS-CoV-2 variants to a certain extent [8–10].

## Conclusion

This work suggested that most of published nAbs with diverse recognizing epitopes still neutralized the SARS-CoV-2_N501Y as similar potencies as that of the wild type virus, except a few of nAbs which targeted the N501 residual directly. Considering the rapid epidemic of several SARS-CoV-2 variants around the world and the significant risk of viral escape, it is crucial to persistently monitor their neutralizing sensibilities to the monoclonal nAbs and sera samples from convalescent COVID-19 patients and vaccine-immunized individuals.

## Supplementary Information

**Additional file 1**. **Figure S1.** Structural depiction of ACE2 (6M0J) and a representative nAb from each class binding to the RBD. Class 1: P2C-1F11, Class 2: P2B-2F6, Class 3: S309, Class 4: EY6A. **Figure S2.** The neutralizations of the nAb panel against the wild type and N501Y mutant SARS-CoV-2 pseudovirus. The neutralizing curves were shown from two independent experiments with similar results. **Figure S3.** The binding affinities of the nAb panel to the wild type and N501Y mutant RBD proteins of SARS-CoV-2 by SPR. (A) The curves were shown from one out of three independent experiments. (B) The data was summarized and shown as mean ± SD (n = 3).

## Data Availability

The data is available and can be used for the academic or research purposes.

## Abbreviations

SARS-CoV-2: Severe acute respiratory syndrome coronavirus 2
nAb: Neutralizing antibody
ACE2: Angiotensin-converting enzyme 2
RBD: Receptor binding domain
SPR: Surface plasmon resonance
WT: Wild type

## References

[1] Shi R, Shan C, Duan X, Chen Z, Liu P, Song J, Song T, Bi X, Han C, Wu L (2020). A human neutralizing antibody targets the receptor binding site of SARS-CoV-2. Nature.

[2] Ju B, Zhang Q, Ge J, Wang R, Sun J, Ge X, Yu J, Shan S, Zhou B, Song S (2020). Human neutralizing antibodies elicited by SARS-CoV-2 infection. Nature.

[3] Hansen J, Baum A, Pascal KE, Russo V, Giordano S, Wloga E, Fulton BO, Yan Y, Koon K, Patel K (2020). Studies in humanized mice and convalescent humans yield a SARS-CoV-2 antibody cocktail. Science.

[4] Zost SJ, Gilchuk P, Case JB, Binshtein E, Chen RE, Nkolola JP, Schafer A, Reidy JX, Trivette A, Nargi RS (2020). Potently neutralizing and protective human antibodies against SARS-CoV-2. Nature.

[5] Kreye J, Reincke SM, Kornau HC, Sanchez-Sendin E, Corman VM, Liu H, Yuan M, Wu NC, Zhu X, Lee CD (2020). A therapeutic non-self-reactive SARS-CoV-2 antibody protects from lung pathology in a COVID-19 hamster model. Cell.

[6] Fratev F: The SARS-CoV-2 S1 spike protein mutation N501Y alters the protein interactions with both hACE2 and human derived antibody: a free energy of perturbation study. *bioRxiv* 2020. 10.1021/acs.jcim.1c01242 34806876

[7] Nelson G, Buzko O, Spilman P, Niazi K, Rabizadeh S, Soon-Shiong P: Impact of South African 501.V2 Variant on SARS-CoV-2 Spike Infectivity and neutralization: a structure-based computational assessment. *bioRxiv* 2021.

[8] Hu J, Peng P, Wang K, Fang L, Luo FY, Jin AS, Liu BZ, Tang N, Huang AL (2021). Emerging SARS-CoV-2 variants reduce neutralization sensitivity to convalescent sera and monoclonal antibodies. Cell Mol Immunol.

[9] Xie X, Liu Y, Liu J, Zhang X, Zou J, Fontes-Garfias CR, Xia H, Swanson KA, Cutler M, Cooper D (2021). Neutralization of SARS-CoV-2 spike 69/70 deletion, E484K and N501Y variants by BNT162b2 vaccine-elicited sera. Nat Med.

[10] Chen RE, Zhang X, Case JB, Winkler ES, Liu Y, VanBlargan LA, Liu J, Errico JM, Xie X, Suryadevara N, et al. Resistance of SARS-CoV-2 variants to neutralization by monoclonal and serum-derived polyclonal antibodies. Nat Med. 2021; 27:717–26. 10.1038/s41591-021-01294-w PMC8058618 33664494

[11] Li Q, Wu J, Nie J, Zhang L, Hao H, Liu S, Zhao C, Zhang Q, Liu H, Nie L (2020). The impact of mutations in SARS-CoV-2 spike on viral infectivity and antigenicity. Cell.

[12] Barnes CO, Jette CA, Abernathy ME, Dam KA, Esswein SR, Gristick HB, Malyutin AG, Sharaf NG, Huey-Tubman KE, Lee YE (2020). SARS-CoV-2 neutralizing antibody structures inform therapeutic strategies. Nature.

[13] Ge J, Wang R, Ju B, Zhang Q, Sun J, Chen P, Zhang S, Tian Y, Shan S, Cheng L (2021). Antibody neutralization of SARS-CoV-2 through ACE2 receptor mimicry. Nat Commun.

[14] Luan B, Wang H, Huynh T: Molecular Mechanism of the N501Y Mutation for Enhanced Binding between SARS-CoV-2’s Spike Protein and Human ACE2 Receptor. *bioRxiv* 2021. 10.1002/1873-3468.14076 PMC8250610 33728680

[15] Du S, Cao Y, Zhu Q, Yu P, Qi F, Wang G, Du X, Bao L, Deng W, Zhu H (2020). Structurally resolved SARS-CoV-2 antibody shows high efficacy in severely infected hamsters and provides a potent cocktail pairing strategy. Cell.

[16] Zhou D, Duyvesteyn HME, Chen CP, Huang CG, Chen TH, Shih SR, Lin YC, Cheng CY, Cheng SH, Huang YC (2020). Structural basis for the neutralization of SARS-CoV-2 by an antibody from a convalescent patient. Nat Struct Mol Biol.

[17] Pinto D, Park YJ, Beltramello M, Walls AC, Tortorici MA, Bianchi S, Jaconi S, Culap K, Zatta F, De Marco A (2020). Cross-neutralization of SARS-CoV-2 by a human monoclonal SARS-CoV antibody. Nature.

[18] Gu H, Chen Q, Yang G, He L, Fan H, Deng YQ, Wang Y, Teng Y, Zhao Z, Cui Y (2020). Adaptation of SARS-CoV-2 in BALB/c mice for testing vaccine efficacy. Science.

[19] Sun S, Gu H, Cao L, Chen Q, Yang G, Li R-T, Fan H, Ye Q, Deng Y-Q, Song X, et al: Characterization and structural basis of a lethal mouse-adapted SARS-CoV-2. *bioRxiv* 2020. 10.1038/s41467-021-25903-x PMC8476561 34580297

[20] Ding R, Wang H, Yang Y, Xie L, Zhang L, Li Q, Liu S, Nie J, Wu J, Qin H (2020). Cross-neutralizing activity of monoclonal antibodies against N501Y mutant strain of SARS-CoV-2. J Appl Virol.

[21] Cao Y, Su B, Guo X, Sun W, Deng Y, Bao L, Zhu Q, Zhang X, Zheng Y, Geng C (2020). Potent neutralizing antibodies against SARS-CoV-2 identified by high-throughput single-cell sequencing of convalescent patients' B cells. Cell.

