# Supplementary material for: Impact of the N501Y substitution of SARS-CoV-2 Spike on neutralizing monoclonal antibodies targeting diverse epitopes
Source: Virol J. 2021 Apr 28;18:87. doi: 10.1186/s12985-021-01554-8 (PMC8081001; doi:10.1186/s12985-021-01554-8)
Supplement: Supplementary file 1 — Additional file 1. Figure S1. Structural depiction of ACE2 (6M0J) and a representative nAb from each class binding to the RBD. Class 1: P2C-1F11, Class 2: P2B-2F6, Class 3: S309, Class 4: EY6A. Figure S2. The neutralizations of the nAb panel against the wild type and N501Y mutant SARS-CoV-2 pseudovirus. The neutralizing curves were shown from two independent experiments with similar results. Figure S3. The binding affinities of the nAb panel to the wild type and N501Y mutant RBD proteins of SARS-CoV-2 by SPR. (A) The curves were shown from one out of three independent experiments. (B) The data was summarized and shown as mean ± SD (n = 3). [file 12985_2021_1554_MOESM1_ESM.docx]

**
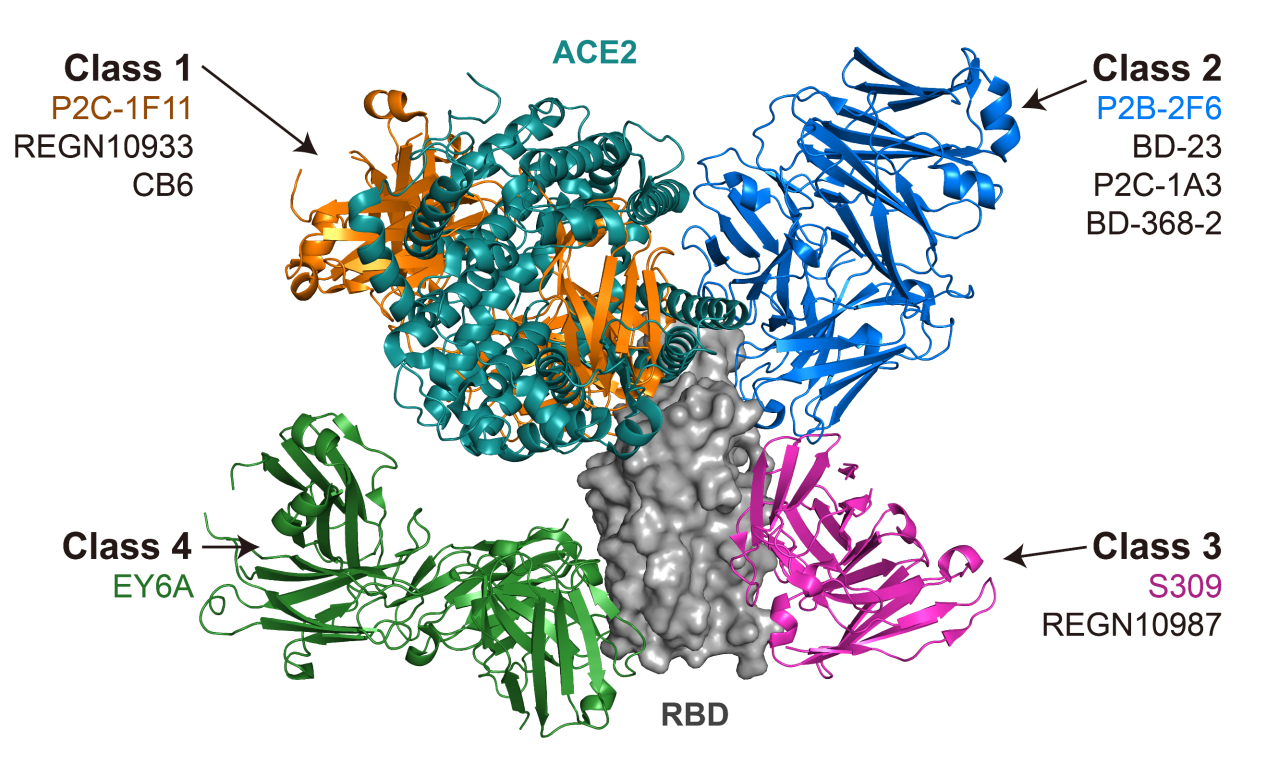
**

**Figure S1.** Structural depiction of ACE2 (6M0J) and a representative nAb from each class binding to the RBD. Class 1: P2C-1F11, Class 2: P2B-2F6, Class 3: S309, Class 4: EY6A.

**
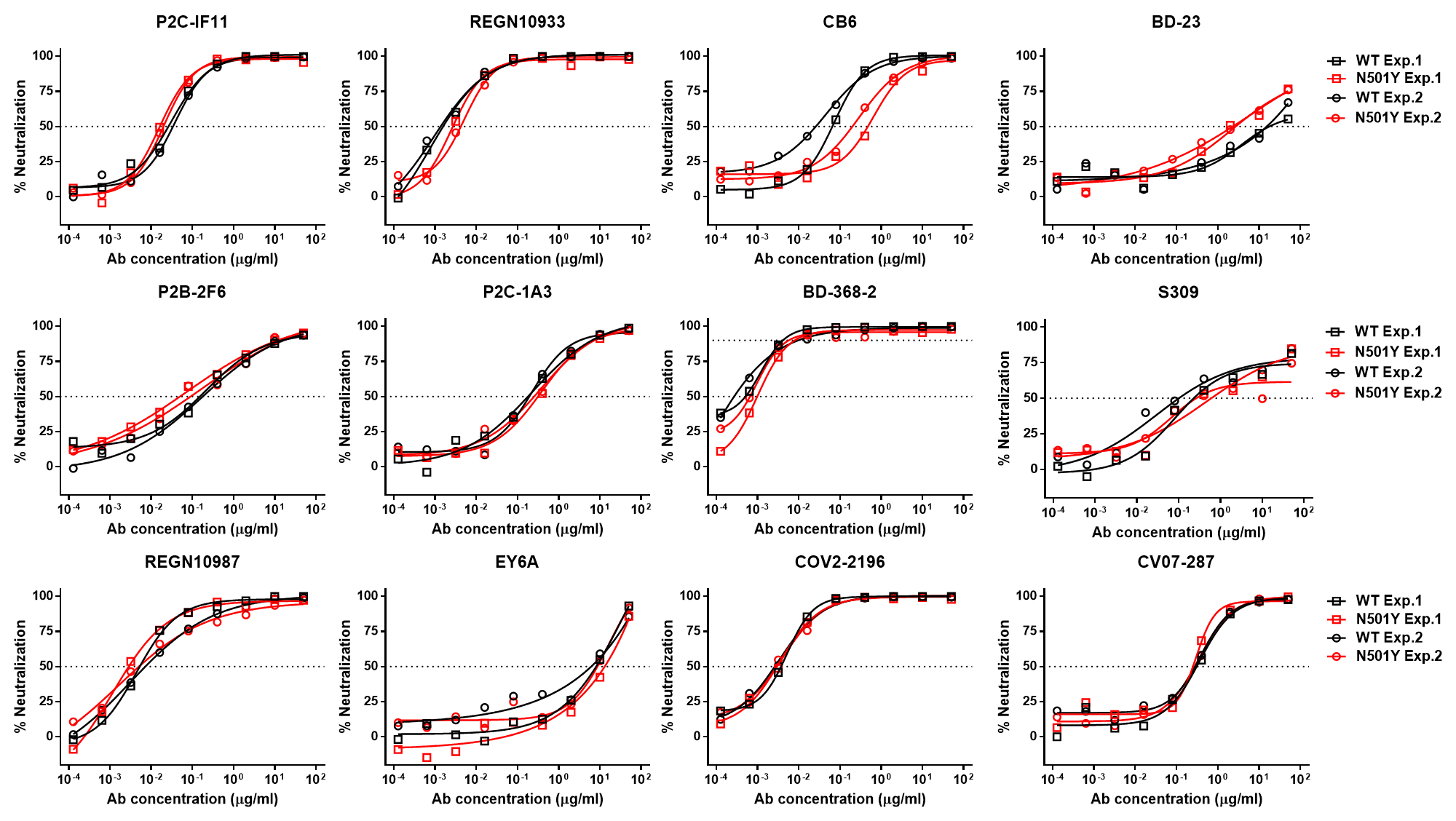
Figure S2. The neutralizations of the nAb panel against the wild type and N501Y mutant SARS-CoV-2 pseudovirus.** The neutralizing curves were shown from two independent experiments with similar results.


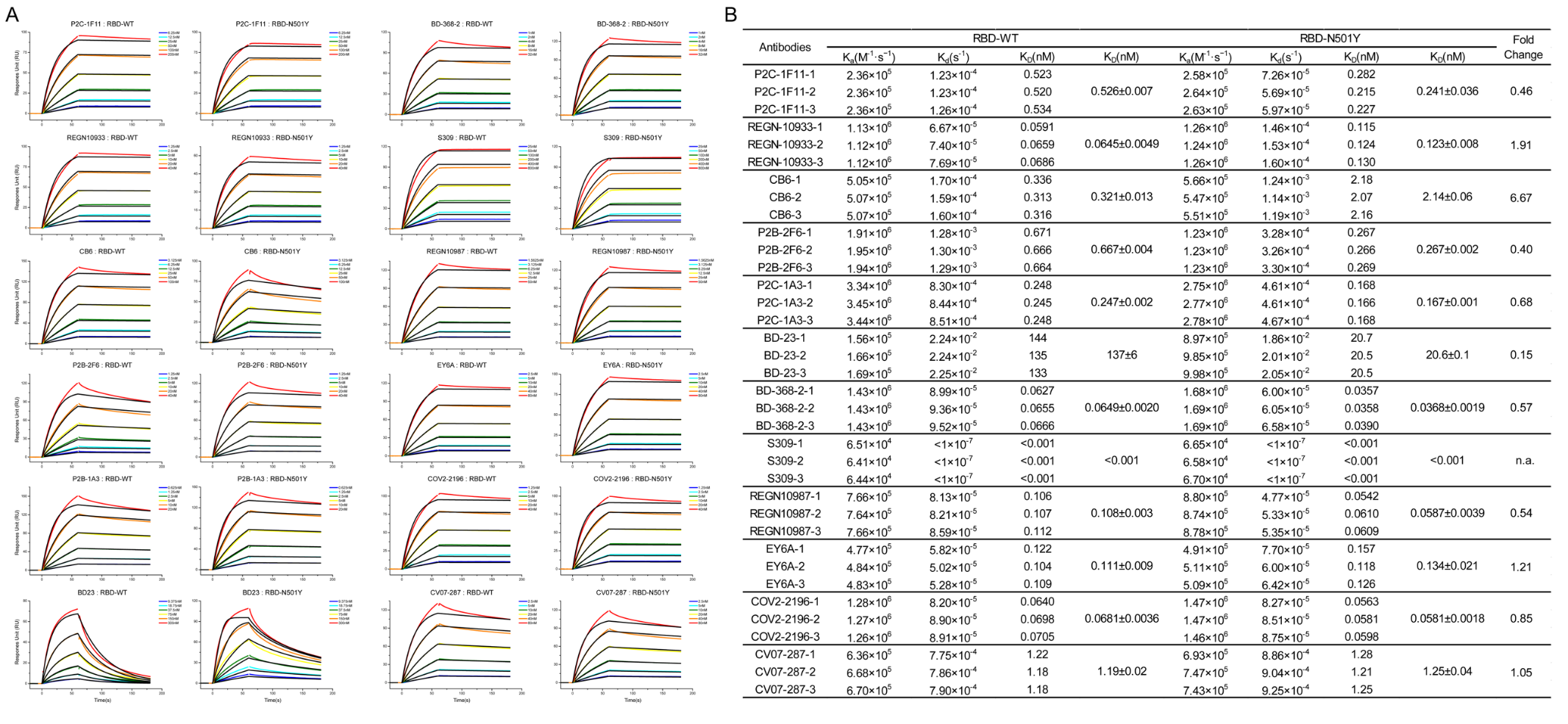


**Figure S3.** **The binding affinities of the nAb panel to the wild type and N501Y mutant RBD proteins of SARS-CoV-2 by SPR.** (A) The curves were shown from one out of three independent experiments. (B) The data was summarized and shown as mean ± SD (n=3).
